# Hydrogen Inhalation Attenuates Oxidative Stress Related Endothelial Cells Injury After Subarachnoid Hemorrhage in Rats

**DOI:** 10.3389/fnins.2019.01441

**Published:** 2020-01-21

**Authors:** Kai Zhuang, Yu-Chun Zuo, Prativa Sherchan, Ji-Kai Wang, Xiao-Xin Yan, Fei Liu

**Affiliations:** ^1^Department of Neurosurgery, The Third Xiangya Hospital, Central South University, Changsha, China; ^2^Department of Neurosurgery, Xiangya Hospital, Central South University, Changsha, China; ^3^Department of Physiology and Pharmacology, School of Medicine, Loma Linda University, Loma Linda, CA, United States; ^4^Department of Anatomy and Neurobiology, Xiangya School of Medicine, Central South University, Changsha, China

**Keywords:** subarachnoid hemorrhage, hydrogen, BBB, NLRP3 inflammasome, microthrombosis, vasospasm

## Abstract

**Background:** Subarachnoid hemorrhage (SAH) is a devastating cerebrovascular disease with poor clinical outcome. Nucleotide binding and oligomerization domain-like receptor family pyrin domain-containing 3 (NLRP3) inflammasome serves a key role in inflammatory response, which may lead to endothelial cell injury and blood-brain barrier (BBB) disruption. Hydrogen (H_2_) is considered a neuroprotective antioxidant. This study was set out to explore whether hydrogen inhalation protects against SAH induced endothelial cell injury, BBB disruption, microthrombosis and vasospasm in rats.

**Methods:** One hundred eighty-two male SD rats were used for the study. SAH was induced by endovascular perforation. H2 at a concentration of 3.3% was inhaled beginning at 0.5 h after SAH for duration of 30, 60 or 120 min, followed by single administration or once daily administration for 3 days. The temporal expression of NLRP3 and ASC in the brain was determined, with the effect of hydrogen inhalation evaluated. In addition, brain water content, oxidative stress markers, inflammasome, apoptotic markers, microthrombosis, and vasospasm were evaluated at 24 or 72 h after SAH.

**Results:** The expression of NLRP3 and ASC were upregulated after SAH associated with elevated expression of MDA, 8-OHdG, 4-HNE, HO-1, TLR4/NF-κB, inflammatory and apoptotic makers. Hydrogen inhalation reduced the expression of these inflammatory and apoptotic makers in the vessels, brain edema, microthrombi formation, and vasospasm in rats with SAH relative to control. Hydrogen inhalation also improved short-term and long-term neurological recovery after SAH.

**Conclusion:** Hydrogen inhalation can ameliorate oxidative stress related endothelial cells injury in the brain and improve neurobehavioral outcomes in rats following SAH. Mechanistically, the above beneficial effects might be related to, at least in part, the inhibition of activation of ROS/NLRP3 axis.

## Introduction

Subarachnoid hemorrhage (SAH) is a life threatening cerebrovascular disease with high morbidity and mortality. SAH mainly affects middle-aged patients and accounts for the highest fatality among all stroke subtypes, which places a huge burden on the economy and society (Suarez et al., 2006; Etminan, 2015). Early brain injury (EBI), a series of pathophysiological changes occurring within the first 72 h after SAH, has been considered a major cause of death and poor outcomes following SAH (Cahill et al., 2006; Caner et al., 2012; Sehba et al., 2012). Additionally, delayed cerebral ischemia (DCI) that occurs 4–14 days after SAH may progress to cerebral infarction and exacerbate brain injury, also causing mortality or severe disability. Recently, microthrombosis due to endothelial cell injury has been implicated as one of the mechanisms that contribute to DCI.

Oxidative stress resulted from oxy-hemoglobin stimulation post SAH is one of the factors involved in EBI (Ayer and Zhang, 2008; Crowley et al., 2008). The imbalance in free radical generation and clearance can lead to oxidative stress, which aggravates inflammatory response and cell death after SAH (Ayer and Zhang, 2008; Ducruet et al., 2010). Thus, anti-oxidative agents are expected to be protective against SAH, and may be a potential promising therapeutic strategy for SAH patients (Wang et al., 2012; Kuo et al., 2013; Zhang et al., 2014).

Inflammation mediated by produced chemokines and cytokines is another major factor responsible for EBI in SAH, (Caner et al., 2012; Chen et al., 2013; Sun et al., 2013). Mounting evidence indicates that NLRP3 inflammasome is a key component of the inflammatory response in various neurological diseases including SAH (Chen et al., 2013; Ma et al., 2014), ischemic stroke (Yang et al., 2014), traumatic brain injury (Liu et al., 2013), and Alzheimer’s disease (Tan et al., 2013). Reactive oxygen species (ROS) generation, K^+^ efflux, Cl^–^ efflux appear to be the three common upstream mediators of NLRP3, while activation of the adapter protein apoptosis-associated speck-like protein containing a CARD (ASC) is also involved (Tang et al., 2017). However, the role and mechanism of NLRP3 inflammasome in the pathogenesis of microthrombosis and vasospasm after SAH remain poorly understood.

Hydrogen (H_2_) is an antioxidant gas that appears to be neuroprotective (Zhan et al., 2012; Zhuang et al., 2012; Zhuang et al., 2013). Recent studies show that hydrogen exerts anti-oxidant, anti-inflammatory, and anti-apoptotic properties. For instance, hydrogen can mitigate ischemia/reperfusion injury of kidney (Cardinal et al., 2010) and intestine (Wu et al., 2017). Hydrogen enriched saline can attenuate neuronal apoptosis and oxidative stress post SAH (Hong et al., 2014). A recent study showed beneficial effects of intraperitoneal injection of hydrogen-rich saline in an experimental SAH model, and hypothesized that the protective effect might involve NLRP3 inflammasome (Shao et al., 2016). Thus, it is necessary to explore whether hydrogen gas inhalation can reduce NLRP3 inflammasome and mitigate this downstream effects after SAH. Given the importance of vascular endothelial injury in the pathophysiology of SAH, the study was aimed to investigate whether hydrogen inhalation can reduce SAH induced microthrombosis and vasospasm via ameliorating endothelial cell inflammation and damage.

## Materials and Methods

### Animals and Surgical Induction of SAH

Adult male Sprague–Dawley rats (250 g–280 g) were purchased from the SLAC Company (Changsha, China). Rats were housed in a room with constant temperature (25°C), humidity control and with a 12/12 h light/dark cycle, with standard animal chow and water provided freely. All the experimental procedures were approved by the Institutional Animal Care and Use Committee of Central South University and performed according to the Guide for the Care and Use of Laboratory Animals of the National Institutes of Health (eighth edition) and the ARRIVE guidelines.

Surgery was performed to induce SAH using a modified endovascular perforation method as previously described (Sugawara et al., 2008). Briefly, rats were anesthetized with pentobarbital (40 mg/kg, i.p.). The left common-, external-, and internal- carotid arteries were exposed, and a 3-centimeter-long sharpened 4–0 monofilament nylon suture was inserted into the left internal carotid artery through the external carotid artery stump until a resistance was felt, and the suture was advanced 3 mm further to perforate the bifurcation of anterior and middle cerebral artery (MCA). Sham-operated rats underwent above identical procedures except for the artery perforation.

### Hydrogen Administration

Hydrogen (H_2_) was administered to experimental rats by inhalation, which was started 0.5 h after the induction of SAH. Hydrogen administration was performed following procedures described in a previous study (Xin et al., 2017). Thus, rats were placed in a transparent chamber that had an inlet connected with 3.3% hydrogen gas inhaler (MIZ, MHG-2000, Japan). The concentration of oxygen in the chamber was maintained at 21% using supplemental oxygen which was monitored continuously with a gas analyzer. Hydrogen concentration in the chamber was maintained at 3.3% with continuous monitoring throughout the duration of inhalation. The animals without hydrogen treatment were placed in a chamber with room air only.

### Animal Grouping and Treatments

**FIGURE 1:**
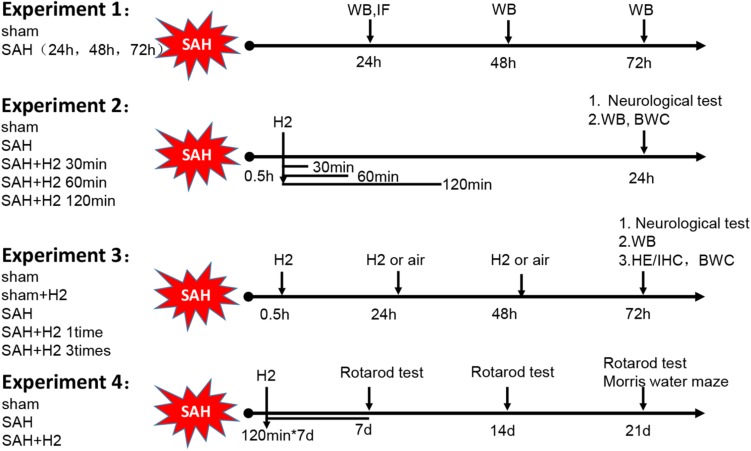
Schematic illustration of experimental design. H2, hydrogen gas; WB, western blot; IF, immunofluorescence; IHC, immunohistochemistry; HE, Hematoxylin Eosin; BWC, Brain water content.

The overall experimental design is shown schematically in Figure 1. The study was divided into four parts. The first part consisted of time course study; the second part was outcome study to evaluate effects of three different durations of H2 inhalation; the third part was outcome study to evaluate the effects of a single or once daily administration of H2 inhalation for 3 days; and the fourth part was to evaluate the long-term outcome after H2 inhalation.

#### Experiment 1

This experiment was designated to determine the temporal expression of NLRP3 and ASC in the ipsilateral left hemisphere at different time-points after SAH. Twenty-four rats were randomly divided into four groups: Sham group surviving 24 h (h) and SAH groups surviving 24, 48, and 72 h post-surgery, respectively (*n* = 6/group). The animals were euthanized at the indicated time-points after SAH, with brain samples analyzed biochemically and histologically.

#### Experiment 2

This experiment was set to evaluate the effect of hydrogen inhalation for 30, 60, and 120 min on the expression of NLRP3 and ASC at 24 h after SAH. Thirty rats were randomly assigned into five groups (*n* = 6/group): Sham, SAH, SAH + H2 (30 min), SAH + H2 (60 min), and SAH + H2 (120 min). Hydrogen gas inhalation was started 0.5 h after SAH, and continued for 30, 60, and 120 min, respectively. Rats in Sham and SAH groups that were supplied with normal room air only. Neurobehavior was evaluated 24 h after surgery. Animals were allowed to survive 24 h, and then the brain samples were collected and subjected to immunoblotting study. Additionally, brain water content was measured in control and H2 treatment groups surviving 120 min, including 18 rats in the Sham, SAH and SAH + H2 groups, respectively (*n* = 6/group). Water contents in neuroanatomical structures were calculated using the formula: Water content (%) = (wet weight – dry weight)/wet weigh × 100%, wherein wet weigh was measured immediately following brain dissection, while dry weight was obtained following 24 h drying of the samples in the oven at 100°C.

#### Experiment 3

This experiment was to determine the effect of one time or three times administration of hydrogen gas inhalation on the expression of NLRP3 and ASC at 72 h after SAH. Thirty rats were randomly assigned to four groups (*n* = 6/group): Sham, Sham + H2, SAH, SAH + H2 (one time) and SAH + H2 (three times). Hydrogen gas inhalation was administered either one time (0.5 h after SAH) or three times (0.5, 24, and 48 h after SAH). Hydrogen gas concentration was maintained at 3.3% and was administered for duration of 120 min each time. Rats in Sham and SAH group were put in the same chamber with normal room air only. Neurobehavior was evaluated 72 h after surgery, followed by brain collection for western blot analysis. Additionally, 24 rats were divided into four groups (*n* = 6/group): Sham, Sham + H2, SAH, SAH + H2 (three times), the brains from these groups of animals were collected for immunohistochemistry. Besides, 18 rats were divided into three groups (*n* = 6/group): Sham, SAH, SAH + H2 (three times), brain water content was measured at 72 h.

#### Experiment 4

This experiment was set to explore the long-term beneficial effect of hydrogen inhalation after SAH. Eighteen rats were randomly assigned to three groups (*n* = 6/group): Sham, SAH and SAH + H2. Hydrogen inhalation was administered for 120 min once daily for 7 days. Rats in Sham and SAH groups served as surgical and vehicle controls, respectively. Neurological scores were evaluated using Rotarod test at 7, 14, and 21 days after SAH and water maze test was performed at 21–25 days after operation.

### Measurement of SAH Grade

Subarachnoid hemorrhage grade was evaluated using a previously established scoring method, with 0–18 points scaling the degree of bleeding (Sugawara et al., 2008), which was carried out by two investigators who were blinded to the experiments. In sham-operated rats, the score was consistently 0. Only the operated animals with SAH grade greater than eight were included in the current study.

### Neurobehavioral Evaluation

Neurobehavioral assessments were performed by an investigator who was blinded to the experiments and animal groups. Modified Garcia test and beam balance test were performed at 24 and 72 h after SAH using as previously described (Sugawara et al., 2008). Rotarod test was performed at days 7, 14, and 21 after SAH to assess sensorimotor coordination and balance. Water maze test was performed at days 21–25 after SAH to evaluate spatial learning and memory as previously described (Lekic et al., 2010; Sherchan et al., 2011).

### Measurement of Lipid Peroxidation

The left hemisphere from freshly removed brain was used for assessment of lipid peroxidation by measurement of malondialdehyde (MDA) using a commercial MDA kits (Cat#S0131, Beyotime, China), following the manufacturer’s instruction. The absorbance of the supernatant was measured by spectrophotometry at 532 nm and quantified using a standard curve. All tests were conducted in triplicate. The MDA concentrations were expressed as fold increase relative to sham group.

### Western Blot Analysis

Western blot was performed as previously described (Xiao et al., 2018). Briefly, proteins were extracted in lysates of the ipsilateral cortex or hippocampus (doublecortin levels) via homogenization using radioimmunoprecipitation (RIPA) buffer (Cat#P0013B, Beyotime, China). The primary antibodies were used with the following dilutions: NLRP3 (1:500, Cat#ab210491, Abcam, United States), ASC (1:500, Cat#sc-51414, Santa Cruz Biotechnology, United States), caspase-1 (1:500, Cat#22915-1-AP, Proteintech, United States), cleaved caspase-1 (1:1000, Cat#ab179515, Abcam, United States), IL-1β (1:500, Cat#16806-1-AP, Proteintech, China), DCX (1:1000, Cat#ab18723, Abcam, United States) TLR4 (1:1000, Cat#ab217274, Abcam, United States), NF-κB(p65) (1:1000, Cat#ab16502, Abcam, United States), phosph-(NF-κB)(p65) (1:1000, Cat#ab106129, Abcam, United States), SOD2 (1:1000, Cat#ab68155, Abcam, United States), 4-HNE (1:1000, Cat#ab46545, Abcam, United States), HO-1 (1:1000, Cat#ab13243, Abcam, United States), Bcl-2 (1:1000, Cat#ab59348, Abcam, United States), Bax (1:1000, Cat#ab32503, Abcam, United States), cleaved caspase-3 (1:500, Cat#9661, Cell Signaling Technology, United States), and GAPDH (1:1000, Cat#10494-1-AP, Proteintech, China).

### Histological, Immunohistochemical and Immunofluorescent Stains

Brain samples were cut into 30 μm thick coronal sections and collected in serial sets as previously described using a cryostat (Leica CM3050S, Buffalo Grove, IL, United States). Consecutive sections from each animal were stained with cresyl violet (Nissl stain), hematoxylin/eosin (HE) and primary antibodies with the avidin-biotin complex (ABC) method or fluorescent-tagged secondary antibodies as previously described (Hu et al., 2017). The primary antibodies used including the following: NLRP3 (1:200, Cat#ab4207, Abcam, United States), ASC (1:200, Cat#sc-51414, Abcam, United States), 8-OHdG (1:200, Cat#ab48508, Abcam, United States), DCX (1:200, Cat#ab18723, Abcam, United States), and fibrinogen (1:500, Cat# LS-B11024, Lifespan, United States). The Nissl stain of the hippocampus was analyzed to assess the neurological damage, the molecular layer and hilus of CA1 were photographed for statistical analysis.

### Co-labeling of Lectin and TUNEL Staining

Lectin and TUNEL co-labeling was performed using a double immunofluorescent method. Briefly, brain sections were incubated with biotinylated lectin (1:500, Cat#B-1175, Vector, United States) followed by AMCA Streptavidin (1:200, Vector, United States) to label vascular endothelium. TUNEL staining was performed using an *in situ* Cell Death Detection Kit (Cat#11684817910, Roche Inc., United States), which was performed following the manufacturer instructions to detect apoptosis as previously described (Xiao et al., 2018). Sections were dehydrated in increasing concentrations of ethanol and cleared in xylenes and then coverslippered. Fluorescently labeled sections were coverslippered with a commercial antifading mounting medium. All sections were examined on a light microscope, with images captured using a built-in imaging system (BX53, Olympus, Japan).

### Statistical Analysis

Sample size was calculated by Sigma Plot, which indicated that the animal numbers *n* = 6/group was deemed sufficient for the experiments with no outliers identified. All measurement data were expressed as mean ± standard derivation (SD). Normal distribution of data was verified with Shapiro–Wilk normality test. One-way analysis of variance (ANOVA) was used in this study to compare differences among groups followed by Tukey’s multiple-comparisons test using the SPSS 18.0 software (SPSS Inc., Chicago, IL, United States). The level of statistically significance difference was set with *p* < 0.05.

## Results

### Animal Number and Mortality Rate

A total of 182 rats were used for the study which comprised of 48 rats in the Sham group and 134 rats in the SAH groups. None of the animals died in the sham group (0 of 48 rats). The overall mortality rate after SAH induction was 17.9% (24 of 134 rats) (Supplementary Figure S1A). There was no significant difference in mortality rates between SAH groups. At 24 h after SAH induction, blood clots were mainly observed around the Circle of Willis and ventral brainstem (Supplementary Figure S1B). There was no significant difference in the average SAH grades among SAH groups with or without hydrogen inhalation (Supplementary Figure S1C).

### SAH Induced Upregulation of NLRP3 and ASC Expression in Ipsilateral Cerebrum

**FIGURE 2:**
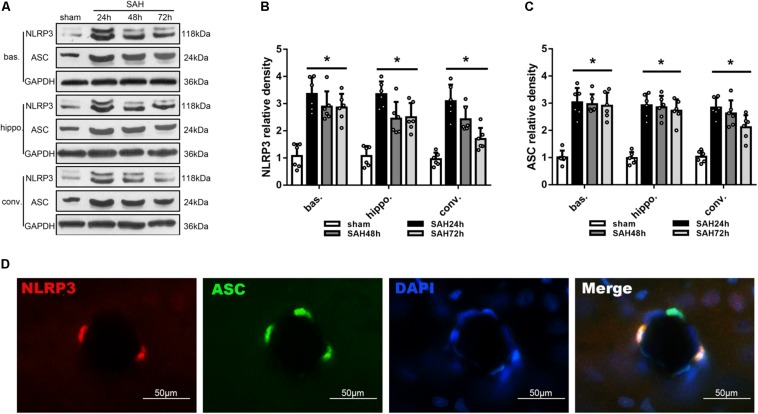
Temporal expression of NLRP3 and ASC in different areas of the ipsilateral hemisphere after unilateral induction of SAH. **(A)** Representative western blots and Quantitative analysis of **(B)** NLRP3 and **(C)** ASC protein expression in the basal cortex (bas.), hippocampus (hippo.), and convex cortex (conv.) at 24, 48, and 72 h after SAH. Data are represented as mean ± SD of relative density. *n* = 6 per group.^∗^*p* < 0.05 vs. sham. **(D)** Double immunofluorescence staining of NLRP3 and ASC. Scale bar = 50 μm.

Modified Garcia score and beam balance test were performed to evaluate the neurological deficit (Supplementary Figures S2A,B). While western blot was performed to determine the protein expression of NLRP3 and ASC in the left basal cortex, hippocampus and convex cortex (Figure 2A) at 24, 48, and 72 h after SAH. Levels of NLRP3 and ASC were peaked at 24 h after SAH and gradually decreased thereafter, although remained significantly elevated up to 72 h after SAH compared to sham group (*p* < 0.05; Figures 2B,C, respectively). Consistent with immunoblotting, double immunofluorescence showed that NLRP3 co-localized ASC in the brain at micro-vessels 24 h after SAH (Figure 2D).

### H2 Inhalation Attenuated NLRP3 and ASC Overexpression, Improved Neurobehavior and Reduced Brain Water Content at 24 h After SAH

**FIGURE 3:**
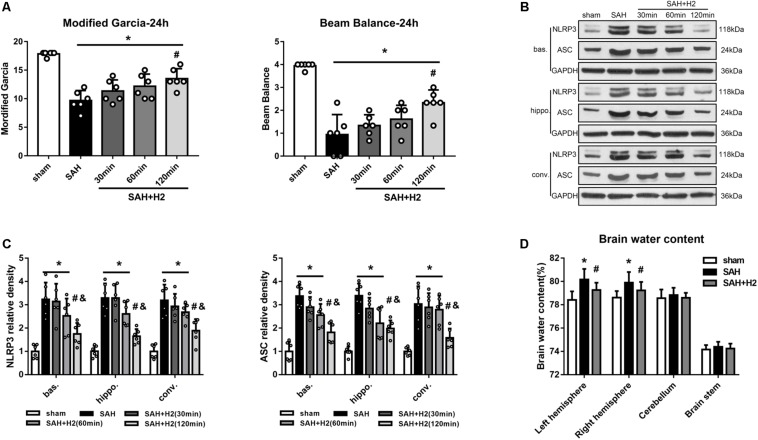
Effect of hydrogen inhalation on neurological scores, NLRP3 and ASC expression and brain edema at 24 h after SAH. **(A)** Modified Garcia score and beam balance test score after hydrogen inhalation treatment for 30, 60, and 120 min duration after SAH. **(B)** Representative western blots and **(C)** quantitative analysis of NLRP3 and ASC protein expression in left basal cortex (bas.), hippocampus (hippo.), and convex cortex (conv.), respectively. **(D)** Brain water content in left hemisphere, right hemisphere, cerebellum and brain stem in sham, SAH and SAH + H2 group. Data represented as mean ± SD. *n* = 6 per group, ^∗^*p* < 0.05 vs. sham; #*p* < 0.05 vs. SAH; &*p* < 0.05 vs. SAH + H2 (30 min).

The modified Garcia score and beam balance score evaluated 24 h after SAH was significantly lower in the SAH groups than sham group, and H2 inhalation for 120 min significantly improved the Garcia score and beam balance neurological score compared with SAH group (*p* < 0.05; Figure 3A). At 24 h after SAH, the expression of NLRP3 and ASC was significantly increased in the left basal cortex, hippocampus and convex cortex (*p* < 0.05; Figures 3B,C). H2 inhalation for 120 min reduced the expression of NLRP3 and ASC in SAH + H2 group when compared with SAH group in the above cerebral subregions (*p* < 0.05; Figures 3B,C). Since H2 inhalation for 120 min was observed to be most effective, we chose 120 min time point inhalation for the next studies. The brain water content in the left hemisphere and right hemisphere were significantly increased in SAH group compared with sham group, while H2 inhalation for 120 min significantly reversed this change in SAH + H2 group compared with SAH group (*p* < 0.05; Figure 3D). However, the brain water content in cerebellum and brain stem show no significantly difference between sham, SAH and SAH + H2 groups (*p* > 0.05; Figure 3D).

### H2 Inhalation Attenuated NLRP3 and ASC Overexpression, Improved Neurobehavior and Reduced Brain Water Content at 72 h After SAH

**FIGURE 4:**
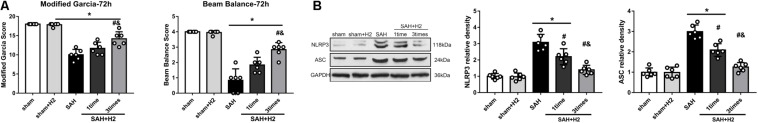
Effect of hydrogen inhalation on neurological scores, NLRP3 and ASC expression and brain edema at 72 h after SAH. **(A)** Modified Garcia score and beam balance test score at 72 h after SAH with hydrogen inhalation single administration (one time) or once daily administration for 3 days (three times). **(B)** Representative western blots and quantitative analysis of NLRP3 and ASC expression in the left hemisphere at 72 h after surgery. Data represented as mean ± SD. *n* = 6 per group, ^∗^*p* < 0.05 vs. sham; #*p* < 0.05 vs. SAH. &*p* < 0.05 vs. SAH + H2 (one time).

The modified Garcia and beam balance scores 72 h after SAH were significantly improved with one time administration and three times administration of hydrogen inhalation for 120 min duration each (*p* < 0.05; Figure 4A). H2 inhalation for three times significantly improved neurological scores 72 h after SAH compared with single administration (*p* < 0.05; Figure 4B). At 72 h after SAH, the expression of NLRP3 and ASC was significantly increased, and H2 inhalation for three times reduced the expression of NLRP3 and ASC as compared with SAH and SAH + H2 (one time) groups (*p* < 0.05; Figure 4B). The brain water content in the left hemisphere and right hemisphere were significantly reduced in SAH+H2 group compared with SAH group (Supplementary Figure S2C). Since H2 inhalation for three times each for 120 min was more effective, we chose hydrogen inhalation three times for the remaining studies.

### H2 Inhalation Ameliorated Microthrombosis and Vasospasm 72 h After SAH

**FIGURE 5:**
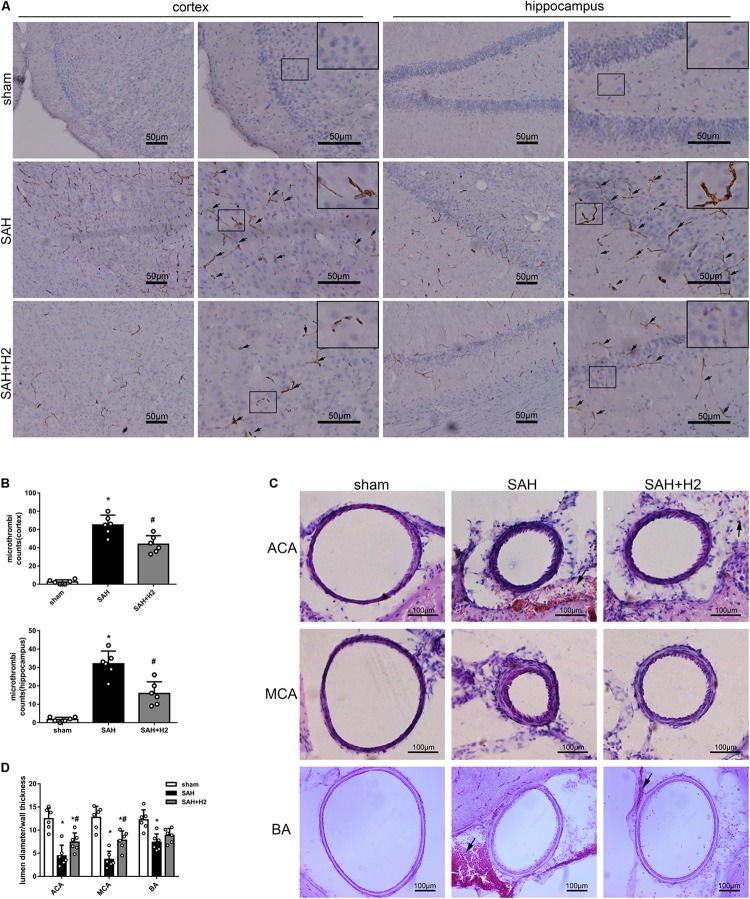
Effect of hydrogen inhalation on microthrombosis and vasospasm at 72 h after SAH. **(A)** Representative images and **(B)** quantitative analysis of microthrombosis in the cortex and hippocampus at 72 h after SAH. Scale bar = 50 μm. **(C)** Representative images and **(D)** quantitative analysis of vasospasm of the anterior cerebral artery (ACA), middle cerebral artery (MCA), and basilar artery (BA) at 72 h after SAH. Scale bar = 100 μm. Data represented as mean ± SD. *n* = 6 per group, ^∗^*p* < 0.05 vs. sham; #*p* < 0.05 vs. SAH.

Immunohistochemistry staining for fibrinogen showed significantly increased microthrombi counts in both cortex and hippocampus after SAH which was reduced with H2 inhalation three times for 120 min each in the SAH + H2 group compared to SAH group (*p* < 0.05; Figures 5A,B). Hematoxylin and eosin staining showed significant vasoconstriction of the anterior cerebral artery (ACA), MCA, and basilar artery (BA) at 72 h after SAH. These changes were reversed in the group with H2 inhalation three times relative to the SAH + H2 group compared to SAH group (*p* < 0.05; Figures 5C,D).

### H2 Inhalation Attenuated TUNEL Positivity and TLR4/NF-κB Activation at 72 h After SAH

**FIGURE 6:**
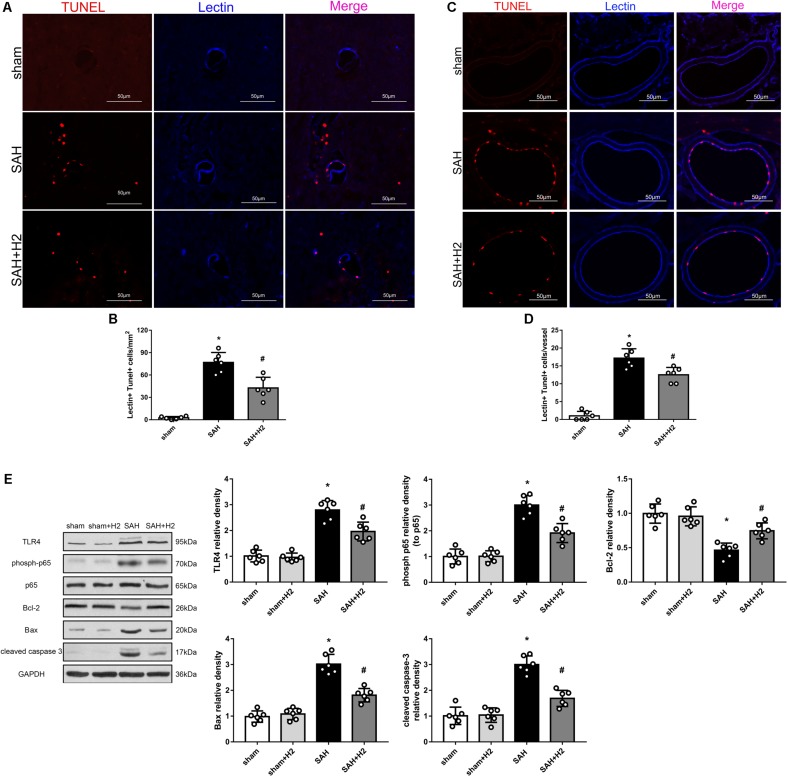
Effect of hydrogen inhalation on endothelium injury in the micro- and macro-vessels at 72 h after SAH. **(A)** Representative immunofluorescence images and **(B)** quantitative analysis of lectin and TUNEL staining in brain micro-vessels. **(C)** Representative images and **(D)** quantitative analysis of lectin and TUNEL staining in brain macro-vessels. **(E)** Representative western blots and quantitative analysis of TLR4, phosoh-p65, Bcl-2, Bax and cleaved caspase-3 in left hemisphere at 72 h. Data represented as mean ± SD. *n* = 6 per group, ^∗^*p* < 0.05 vs. sham; #*p* < 0.05 vs. SAH.

Double staining of lectin and TUNEL staining showed increased TUNEL positive cells in the micro-vessels 72 h after SAH, which was reduced in the SAH + H2 group compared to SAH group (Figures 6A,B). Similarly, TUNEL positive cells in the macro-vessels was reduced in the SAH + H2 group compared to SAH group (Figures 6C,D). Given that oxidative stress may mediate DNA damage via TLR4/NF-κB pathway, we tested if H2 inhalation could affect TLR4 pathway. Immunoblotting data indicated that levels of TLR4 and phospho-NF-κB (p65), Bax and cleaved caspase-3 were significantly increased in SAH group compared with sham group, whereas the Bcl-2 was significantly reduced. After H2 inhalation, there was a trend of reversal of above changes (*p* < 0.05; Figure 6E).

### H2 Inhalation Improved Long-Term Neurological Outcome After SAH

**FIGURE 7:**
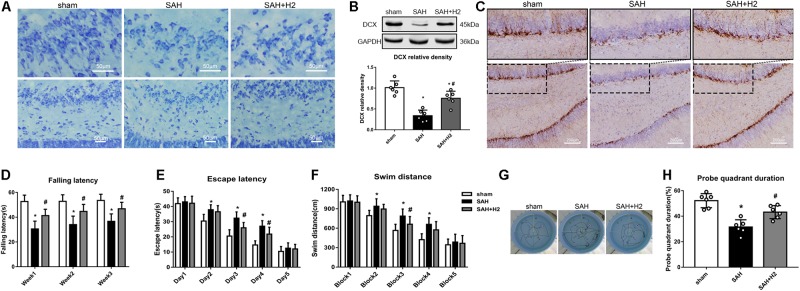
Effect of hydrogen inhalation on long term neurobehavior after SAH. **(A)** Representative images of Nissl staining in the hippocampus. **(B)** Representative western blot images and quantitative analysis of doublecortin (DCX) expression. **(C)** Immunohistochemistry images of DCX in the hippocampus. **(D)** Rotarod test performed at weeks 1, 2, and 3 after SAH. **(E)** Escape latency and **(F)** Swim distance and **(G)** Representative trace images of water maze test performed at 3 weeks after SAH. **(H)** Probe quadrant duration in the probe trial test. Data represented as mean ± SD. *n* = 6 per group. ^∗^*p* < 0.05 vs. sham group; #*p* < 0.05 vs. SAH group.

Memory and cognitive function deficit was related to the hippocampal injury after SAH. Nissl staining showed that H2 inhalation ameliorated neuronal injury in the hippocampus after SAH (Figure 7A). Specifically, the expression of doublecortin (DCX), a marker for immature neurons, was reduced after SAH and H2 inhalation reversed this change (Figures 7B,C, respectively). In Rotarod test, SAH group had a significantly shorter latency to fall compared with the sham group at 1, 2, and 3 weeks after SAH. H2 inhalation daily 120 min for 7 days improved the latency to fall in the SAH + H2 group compared to SAH group (*p* < 0.05; Figure 7D). In the Water Maze test, the escape latency and swim distance traveled to find the platform was longer in the SAH group compared to sham group. However, H2 inhalation significantly reduced escape latency on days 3 and 4 of testing and decreased swim distance during block three of testing in the SAH + H2 group compared to SAH group (*p* < 0.05; Figures 7E–G). In the probe trial, SAH group spent less time in the target quadrant when the platform was removed compared with sham group (*p* < 0.05; Figure 7H). Again, H2 inhalation improved the duration spent in probe quadrant in SAH + H2 group compared to SAH group.

### H2 Inhalation Attenuated Oxidative Stress 72 h After SAH

**FIGURE 8:**
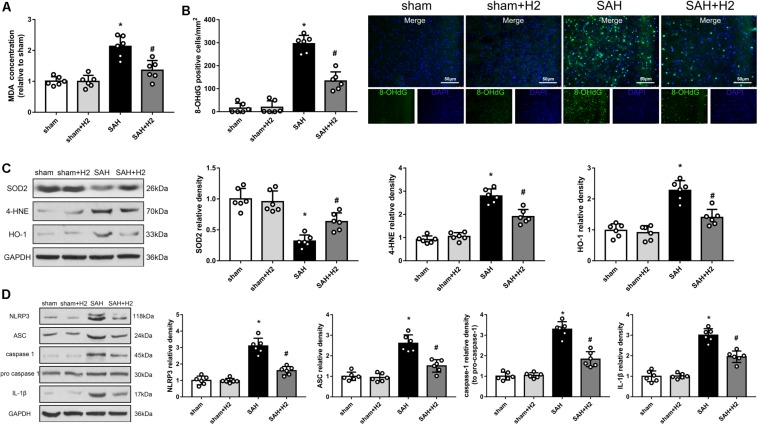
Modulation of hydrogen inhalation on ROS/NLRP3 axis. **(A)** The concentration of MDA in different groups. **(B)** Quantitative analysis and representative images of 8-OHdG staining at 72 h after SAH. **(C)** Representative western blots and quantitative analysis of SOD2, 4-HNE, HO-1 in the left hemisphere 72 h after surgery. **(D)** Representative western blots and quantitative analysis of NLRP3, ASC, cleaved caspase-1, caspase-1, and IL-1β in the left hemisphere. Data represented as mean ± SD. *n* = 6 per group, ^∗^*p* < 0.05 vs. sham; #*p* < 0.05 vs. SAH.

Oxidative stress induced by SAH was considered as one of main cause of EBI, and may affect multiple cellular elements including endothelial cells. Lipid peroxidation reflected by the concentration of MDA and 8-OHdG may relate to oxidative stress induced DNA damage. We detected changes in the concentration of MDA (Figure 8A), 8-OHdG staining (Figure 8B) as well as the levels of SOD2, 4-HNE and HO-1 (Figure 8C) in the brain at 72 h after SAH. Thus, MDA concentration, 8-OHdG positive cells and the expression of 4-HNE and HO-1 were increased, while the expression of anti-oxidant protein SOD2 was decreased. Notably, these changes were reversed by hydrogen inhalation compared to SAH group.

### H2 Inhalation Ameliorated NLRP3 Inflammasome Activation 72 h After SAH

The NLRP3 inflammasome is assembled when the cell is under oxidative stress, and the ROS directly can induce this assembly. Therefore, we speculated that H2 inhalation could reduce endothelial cells injury through inhibit NLRP3 inflammasome. As expected, levels of NLRP3, ASC, cleaved caspase-1, and IL-1β were increased in SAH group compared with the sham group (*p* < 0.05; Figure 8D). H2 inhalation reduced the expression of NLRP3, ASC, cleaved caspase-1, and IL-1β in SAH + H2 group compared with SAH group (*p* < 0.05; Figure 8D).

## Discussion

The present study extended evidence in support of a neuroprotective effect of hydrogen therapy for hemorrhagic cerebral stroke. Major findings of this study include (1) NLRP3 and ASC was upregulated after SAH with highest level noted at 24 h after SAH in the basal cortex, hippocampus and convex cortex; (2) H2 inhalation downregulated the expression of NLRP3 inflammasome proteins and improved neurobehavioral function after SAH. (3) H2 inhalation attenuated microthrombosis and vasospasm after SAH. (4) H2 inhalation improved long-term neurological function after SAH. (5) H2 inhalation reduced oxidative stress partially related to ROS/NLRP3 inflammasome axis.

Reactive oxygen species generated by mitochondrial dysfunction play a significant role in oxidative stress to various types of cells in the brain including microglia, astrocytes, neurons, and endothelial cells, which has been related to inflammation, apoptosis and neurological deficits after SAH (Ayer and Zhang, 2008; Sorce and Krause, 2009; Skowronska and Albrecht, 2013; Sahebkar et al., 2018; Su et al., 2018). The NLRP3 inflammasome composed of NLRP3, ASC and caspase-1, is responsible for the maturation and secretion of proinflammatory cytokines IL-1β and IL-18. A recent study showed that mitochondrial ROS, K^+^ efflux, Cl^–^ efflux are essential and proximal upstream factors for NLRP3 inflammasome assembly (Tang et al., 2017). Inhibition of NLRP3 inflammasome formation has protective effect after SAH (Chen et al., 2013; Zhou et al., 2018). In this study, we measured NLRP3 and ASC expression in different brain regions after SAH including the left hippocampus, convex cortex and basal cortex samples. We observed NLRP3 upregulation in all these regions, indicating that SAH could induce mitochondrial ROS affected broad brain regions.

Hydrogen has been considered a therapeutic anti-oxidative agent for decades as it can easily penetrate the blood brain barrier via gaseous diffusion. Considering its explosive potential (safe concentration between 2 and 5%) (Zhan et al., 2012), we used 3.3% as the dosage in the current study. Previous study reported that hydrogen applied at 1 h after injury ameliorated oxidative stress and showed neuroprotective effect at 24 h, but failed to show beneficial effect at 72 h after SAH (Zhan et al., 2012). This suggests that the therapeutic window and treatment duration are important when designing hydrogen therapy. Hydrogen inhalation was started 30 min after SAH in our study, with different duration of hydrogen inhalation, single vs. multiple times inhalation also tested to determine the optimal regime for hydrogen inhalation. Our results suggest that hydrogen inhalation for 120 min once daily for 3 and 7 days can improve short-term and long-term neurobehavior outcomes after SAH.

Vasospasm has been considered as the main pathophysiological cause of delayed brain injury. Recently, microthrombosis has been shown to play a major role in DCI (Vergouwen et al., 2008). Indeed, autopsy studies observed ischemic regions rich of microthrombi, while vasospasm was neither necessary nor sufficient to induce delayed ischemic deficits (Suzuki et al., 1990; Vergouwen et al., 2008). Endothelial cell death and coagulation induced by inflammation have been well established (Muroi et al., 2014; Frontera et al., 2017), and considered the main cause of microthrombosis (Sabri et al., 2012). We found co-labeling of the endothelial marker lectin and TUNEL, indicating DNA damage in endothelial cells after SAH. We also observed NLRP3 expression in the endothelial cells after SAH suggestive of endothelial inflammation. Interestingly, we found that both microvessels and macrovessels were affected after SAH, and hydrogen inhalation could ameliorate these changes in the vascular profiles.

Oxidative stress to lipids and proteins are a part of the pathophysiology of SAH and can result in cellular damage and dysfunction (Facchinetti et al., 1998; Zhan et al., 2012). Elevation of MDA and 8-OHdG are reported after SAH (Mo et al., 2018). In this study we also observed increased levels of MDA and 8-OHdG in the brain after SAH, and hydrogen inhalation significantly reversed these changes. Therefore, hydrogen inhalation can provide antioxidative protective effect on lipids and proteins after SAH.

Inflammation is a significant causal factor in EBI after SAH, as suggested by evidence of the involvement of NLRP3 inflammasome and TLR4/NF-κB in SAH (Kawakita et al., 2017; Zhou et al., 2018), which may related to the early as well as late neuronal injuries (Zhou et al., 2007; You et al., 2013; Kawakita et al., 2017; Okada and Suzuki, 2017). Other study showed that the regulation of TLR4/NF-κB pathway by melatonin alleviated secondary brain damage and neurobehavioral dysfunction in SAH model (Wang et al., 2013). Recently, hydrogen rich saline was reported to attenuate EBI by repressing NLRP3 and NF-κB related inflammation (Shao et al., 2016). Consistently, in this study we observed that hydrogen inhalation decreased levels of NLRP3 inflammasome and the TLR4/NF-κB pathway proteins after SAH.

Cellular damage including apoptosis is associated with EBI in cerebral stroke (Hasegawa et al., 2011). Specifically, endothelial cells play an essential role in maintaining the blood brain barrier, while endothelial dysfunction contributes to vascular disease and stroke (Roquer et al., 2009). Inflammation and apoptosis can induce endothelial damage after stroke (Suzuki and Urano, 2011; Xu et al., 2017). Our results showed that NLRP3 in the endothelial cells after SAH could be mitigated by hydrogen inhalation. We also showed a beneficial effect of hydrogen inhalation in antagonizing DNA fragmentation as indicated by TUNEL positivity in vascular cells induced by experimental SAH. Moreover, we also observed that immature neurons in the hippocampal dentate gyrus were reduced after SAH, and hydrogen inhalation attenuated damage to immature neurons.

Previous studies have shown that hydrogen therapy could improve neurological outcome in animal models of ischemic and hemorrhagic cerebral stroke (Zhan et al., 2012; Zhuang et al., 2012; Shao et al., 2016; Wang et al., 2016; Ono et al., 2017). Hydrogen inhalation can attenuate postoperative cognitive impairment by regulating inflammation and apoptosis in a rat model (Xin et al., 2017). However, long-term outcomes following hydrogen inhalation after SAH has not been previously studied. In this study, we observed that hydrogen inhalation daily for 7 days after SAH improved long-term neurological function evaluated by Rotarod test and water maze test in SAH rats. Consistently, we observed that hydrogen inhalation decreased inflammation and apoptosis markers after SAH which was associated with reduced vasospasm, microthrombosis, cellular injury and improved neurological function after SAH in rats.

Hydrogen products including hydrogen gas and hydrogen saline have broad applications for brain injuries derived from ischemia/reperfusion (Cui et al., 2016; Wang et al., 2016; Yu et al., 2017) and hemorrhage (Hong et al., 2014) in both animal models (Li et al., 2015) and clinical patients (Ono et al., 2017). There are several advantages of hydrogen gas application for clinical use. First, inhalation is a convenient method for hydrogen administration in the clinical setting. Moreover, hydrogen gas is stable without restraints by temperature and humidity. However, there are some limitations in the present study. For example, *in vitro* experiments would be useful to verify inhibition of NLRP3 inflammasome in endothelial cells by hydrogen is relevant to its overall neuroprotective effects after SAH. Further studies are also needed to elucidate the mechanisms by which hydrogen inhalation ameliorates microthrombosis and vasospasm. Moreover, while our findings support an involvement of TLR4/NF-κB pathway in the brain injury after SAH (Wang et al., 2013; Shao et al., 2016), the link of this signaling to hydrogen remains to be explore in detail. Nonetheless, the data presented in the current study support that hydrogen is a promising therapy in clinical management of cerebral strokes.

## Conclusion

Based on the findings in the current study, we conclude that hydrogen inhalation can improve neurobehavioral outcomes after SAH, which may be related to, at least in part, the effect of this gas in ameliorating microthrombosis and vasospasm, NLRP3 inflammasome formation, activation of the TLR4/NF-κB pathway, and DNA damage in endothelial cells.

## Data Availability Statement

All datasets generated for this study are included in the article/Supplementary Material.

## Ethics Statement

The animal study was reviewed and approved by the Institutional Animal Care and Use Committee of Central South University.

## Author Contributions

KZ, Y-CZ, FL, and X-XY participated in the experimental design and manuscript preparation. KZ, Y-CZ, and J-KW performed the experiments. KZ and J-KW collected and analyzed the data. Y-CZ, X-XY, and FL interpreted the data. KZ and Y-CZ drafted the manuscript. PS revised the manuscript and proofread the language. All authors read and approved the final manuscript.

## Conflict of Interest

The authors declare that the research was conducted in the absence of any commercial or financial relationships that could be construed as a potential conflict of interest.

## Supplementary Material

FIGURE S1 **(A)** The number of rats used in each group and mortality. WB, western blot; IF, immunofluorescence; IHC, immunohistochemistry; BWC, Brain water content. **(B)** Representative images of the brain samples from each group at 24 h after surgery. Subarachnoid blood clots were observed mainly around the Circle of Willis and ventral brainstem. No blood was present in the sham group. **(C)** The SAH grade in sham, SAH, and SAH + H2 group.

FIGURE S2 **(A)** Modified Garcia score and **(B)** beam balance test score at 24, 48, and 72 h after SAH. **(C)** Brain water content in left hemisphere, right hemisphere, cerebellum and brain stem in sham, SAH and SAH + H2 group at 72 h. Data represented as mean ± SD. *n* = 6 per group, ^∗^*p* < 0.05 vs. sham; #*p* < 0.05 vs. SAH.
