# Supplementary figures and images for: Hydrogen Inhalation Attenuates Oxidative Stress Related Endothelial Cells Injury After Subarachnoid Hemorrhage in Rats
Source: Front Neurosci. 2020 Jan 21;13:1441. doi: 10.3389/fnins.2019.01441 (PMC6985445; doi:10.3389/fnins.2019.01441)

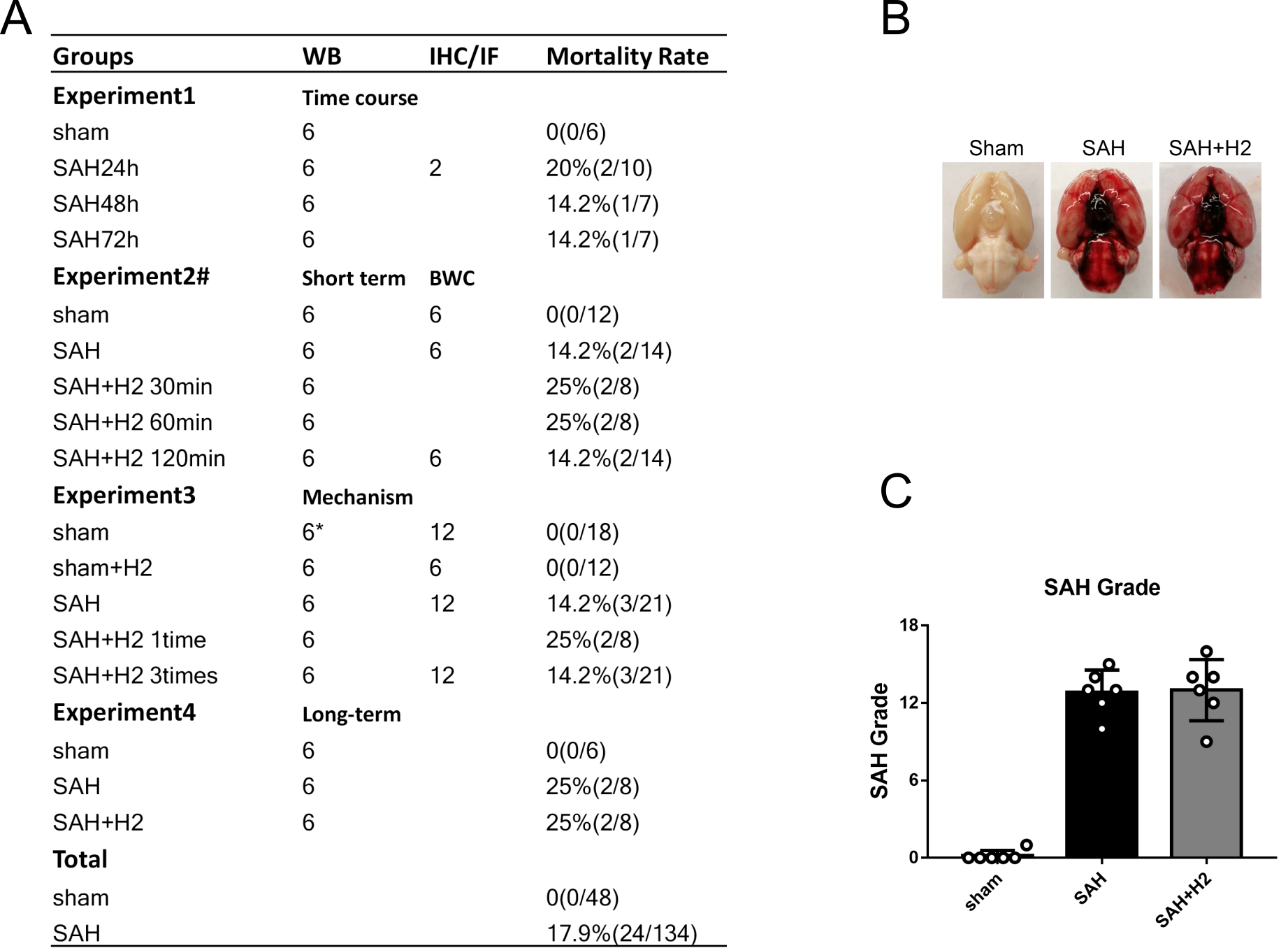

Supplement: FIGURE S1 — (A) The number of rats used in each group and mortality. WB, western blot; IF, immunofluorescence; IHC, immunohistochemistry; BWC, Brain water content. (B) Representative images of the brain samples from each group at 24 h after surgery. Subarachnoid blood clots were observed mainly around the Circle of Willis and ventral brainstem. No blood was present in the sham group. (C) The SAH grade in sham, SAH, and SAH + H2 group. [file Image_1.TIF]

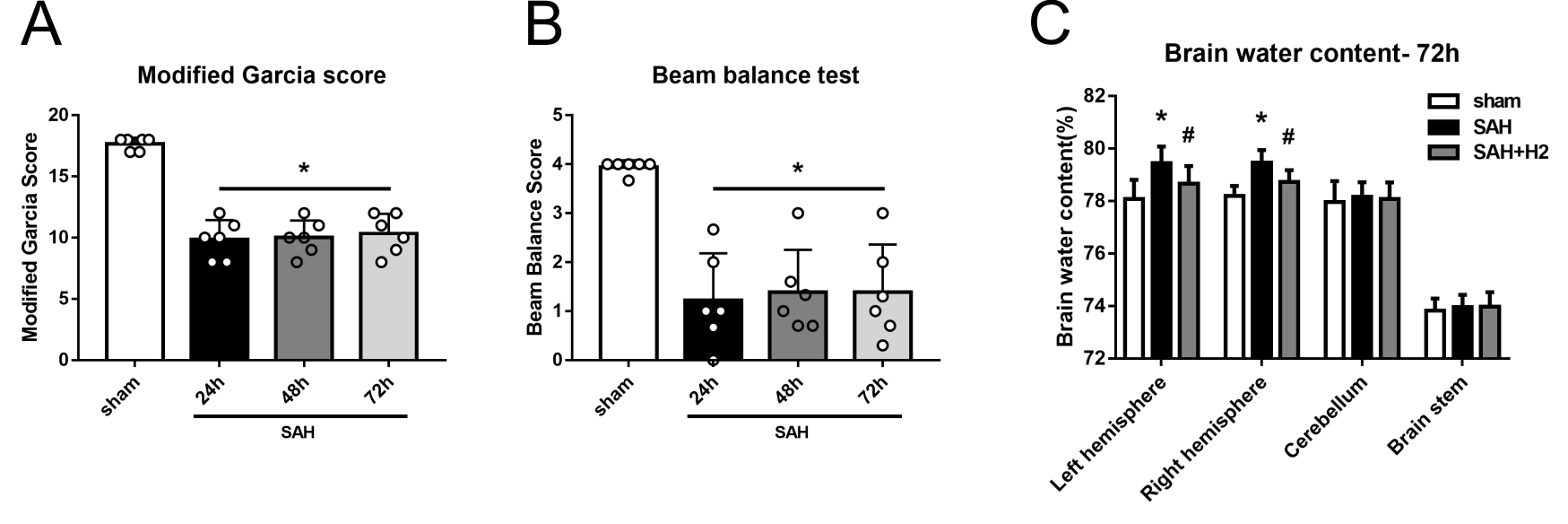

Supplement: FIGURE S2 — (A) Modified Garcia score and (B) beam balance test score at 24, 48, and 72 h after SAH. (C) Brain water content in left hemisphere, right hemisphere, cerebellum and brain stem in sham, SAH and SAH + H2 group at 72 h. Data represented as mean ± SD. n = 6 per group, ∗p < 0.05 vs. sham; #p < 0.05 vs. SAH. [file Image_2.TIF]
